# Metabolomic analysis of seminal plasma to identify goat semen freezability markers

**DOI:** 10.3389/fvets.2023.1132373

**Published:** 2023-03-10

**Authors:** Bingbing Xu, Xue Bai, Jian Zhang, Boyuan Li, Yanjun Zhang, Rui Su, Ruijun Wang, Zhiying Wang, Qi Lv, Jiaxin Zhang, Jinquan Li

**Affiliations:** ^1^College of Animal Science, Inner Mongolia Agricultural University, Hohhot, China; ^2^Key Laboratory of Animal Genetics, Breeding and Reproduction, Hohhot, China; ^3^Key Laboratory of Mutton Sheep Genetics and Breeding, Ministry of Agriculture and Rural Affairs, Hohhot, China; ^4^Inner Mongolia Jinlai Animal Husbandry Technology Co., Ltd., Hohhot, China

**Keywords:** metabolomic, goat spermatozoa, cryopreservation, freezability, seminal plasma

## Abstract

Factors affecting sperm freezability in goat seminal plasma were investigated. Based on the total motility of thawed sperm, goats were divided into a high-freezability (HF) group with >60% total motility (*n* = 8) and a low-freezability (LF) group with <45% total motility (*n* = 8). Sperm and seminal plasma from the HF and LF groups were separated, HF seminal plasma was mixed with LF spermatozoa, LF seminal plasma was mixed with HF sperm, and the products were subjected to a freeze-thaw procedure. Semen from individual goats exhibited differences in freezability. HF semen had higher sperm motility parameters and plasma membrane and acrosome integrity after thawing; this difference could be related to the composition of seminal plasma. Seminal plasma from the HF and LF groups was evaluated using metabolomic analysis, and multivariate statistical analysis revealed a clear separation of metabolic patterns in the seminal plasma of goats with different freezability classifications. Forty-one differential metabolites were identified using the following screening conditions: variable importance in the projection > 1 and 0.05 < *P*-value < 0.1. Kyoto Encyclopedia of Genes and Genomes pathway analysis revealed significant enrichment of central carbon metabolism in cancer, protein digestion and absorption, aminoacyl-tRNA, and other pathways and significant differences in the abundance of seven differential metabolites, including L-glutamine, L-aspartate, L-arginine, phenylpyruvate, benzoic acid, ketoisocaproic acid, and choline between seminal plasma from the HF and LF groups (*P*-value < 0.05). These significantly differentially-expressed metabolites may be potential biomarkers for sperm freezability. L-glutamine, L-aspartate, and L-arginine may directly affect sperm freezability. Benzoic acid, ketoisocaproic acid, and choline may regulate sperm freezability by participating in anabolic processes involving phenylalanine, leucine, and phosphatidylcholine in sperm.

## 1. Introduction

Seminal plasma is a mixture of testicular, epididymal, and accessory sexual gland secretions in male animals. Seminal plasma contains many organic and inorganic components required for sperm function, such as amino acids, proteins, ions, sugars, and lipids, and it is an important source of energy and a transport medium for sperm (1, 2). Seminal plasma is involved in regulating important biological processes in sperm, such as hyperactivation motility, the acrosome reaction, and capacitation (3–6), but its role in semen cryopreservation is difficult to determine because of the complexity of its components.

Several studies have described conflicting views of the effects of seminal plasma on sperm function after cryopreservation. According to a study by Moore et al., exposure of equine spermatozoa to seminal plasma had no significant effect on sperm motility after cryopreservation, but prior to the freezing process, prolonged exposure of spermatozoa to seminal plasma was detrimental (7). Seminal plasma improved the progressive motility and cell structural integrity of boar sperm during cryopreservation (8). The functional variation of seminal plasma after spermatozoa cryopreservation in various species may be attributed to differences in seminal plasma composition caused by the anatomical structure of the accessory sexual glands. However, differences in the contribution of seminal plasma to sperm cryopreservation have also been found in the same species. Rickard found that sperm from low-resilience rams frozen with seminal plasma from high-resilience rams exhibited higher viability after thawing than their own sperm plasma (9). Supplementation of autologous or homologous seminal plasma with high cryoresistance significantly improved sperm fertility and the average number of sperm cells bound to the zona pellucida in stallions (10, 11). These discrepancies were attributed to significant variations in the composition and abundance of small molecular metabolites in seminal plasma.

The metabolome, which is composed of the end products of metabolism within a biological system, determines the current phenotypic state of the cell, and metabolomic analysis is a promising tool for correlating metabolites with cellular biological functions (12, 13). Metabolites have been identified in bovine seminal plasma using metabolomic analysis, and the potential of these metabolites as biomarkers of bovine fertility has been explored (14–16). In this study, we investigated the factors of seminal plasma that affect variation of goat semen freezability and analyzed the metabolites of goat seminal plasma with different freezability classifications using metabolomic analysis. This study aimed to determine whether the seminal plasma metabolome can provide new insights into regulation of the effects of cryopreservation. Identification of potential markers in seminal plasma will provide new suggestions for optimizing existing goat semen cryopreservation strategies.

## 2. Materials and methods

### 2.1. Animals and experimental design

All chemicals used in this study, except for antibiotics (Gibco, USA), were purchased from Sigma-Aldrich (St. Louis, MO, USA). Semen samples from twenty bucks were provided by Inner Mongolia JinLai Livestock Technology Co., Ltd. (TuZuo country, Hohhot, Inner Mongolia, China). All bucks with normal ferility, and housed in the same nutrition and management conditions. Semen was collected with an artificial vagina, and the semen was collected three times a week during the reproductive season. To eliminate the influence of the experimental method on the quality of thawed sperm, we applied the same freezing dilution to semen from different individuals and performed a uniform cryopreservation procedure, which ensured maximum accuracy of the experiments. Three experiments were carried out to characterize individual variation of goat semen freezability and to determine whether this variation could be attributed to seminal plasma. First, spermatozoa were assessed to identify bucks with consistently high or low freezability (HF or LF, respectively). Second, mixing of high-freezability seminal plasma (HSP) with LF sperm as EX-LF group and low-freezability seminal plasma (LSP) with HF sperm as EX-HF group, which were performed before a freeze-thaw procedure. Finally, a metabolomic analysis was performed on seminal plasma with different freezability classifications.

### 2.2. Semen handling

Ejaculates from each buck were centrifuged at 600 × g for 15 mins at 4°C. The supernatant was aspirated and subjected to further centrifugation (10,000 × g, 30 mins) to remove any remaining sperm and cell debris. Then, goat seminal plasma was stored at −80°C; one aliquot was used for remixing semen, and another aliquot was used for further metabolomic analysis.

Ejaculates were slowly diluted with a Tris-citrate-glucose diluent (1:1, semen:diluent, v/v). Centrifugation (200 × g, 27°C, 5 mins) was performed to gently wash the aliquots with the Tris-citrate-glucose diluent. The supernatant, which contained seminal plasma, diluent, and other contaminants, was discarded, and the washed sperm pellets were collected. According to the experimental design described in 2.1, spermatozoa identified as having HF or LF were pooled with HSP or LSP. Washed spermatozoa were diluted (1:10) with a freezing extender that contained 12% seminal plasma. The freezing diluent consisted of 300 mM Tris, 95 mM citric acid, 56 mM glucose, 10% (v/v) egg yolk, 5% glycerol (v/v), and 1% antibiotics (Gibco) (v/v). Ejaculates were slowly diluted (1:10, semen: diluent, v/v) with freezing diluent.

### 2.3. Semen cryopresevation

The concentration of each sample was determined using a Bovine Accuread Photometer (IMV, France) before being diluted to a concentration of 2 × 10^8^ spermatozoa/mL. Samples were then chilled to 4°C for 3 h, and then 200 mL of the sample was stored in 0.25 mL straws (IMV). The straws were placed at equal intervals on a pre-cooled freezing rack before exposure to liquid nitrogen vapor (4 cm above the liquid nitrogen surface for 7 mins). All straws were then submerged in liquid nitrogen and stored. After a week of storage, the straws were thawed in a water bath at 37°C for 30 s.

### 2.4. Evaluation of sperm motility variables

Sperm motility was evaluated after thawing. A 3 μL drop of thawed sperm was placed on a pre-warmed Leja slide analysis chamber, and a computer-assisted sperm analysis system was used (CASA, IVOS II, IMV). A minimum of five fields were evaluated per sample, with a total of 1,000 spermatozoa counted. The sperm motion measurements mainly included total motility (TMOT; %), progressive motility (PMOT; %), curvilinear velocity (VCL; μm/s), straight line velocity (VSL; μm/s); average path velocity (VAP; μm/s), linearity (LIN, %), and amplitude of lateral head displacement (ALH; μm) were evaluated in fresh and frozen/thawed samples. Definitions of these sperm motility parameters can be found in Dorado (17).

### 2.5. Measurement of sperm cell structural integrity

Flow cytometry was performed using an ACEA NovoCyteTM flow cytometer (ACEA, China). To ensure stability of the results, the flow cytometer was outfitted with a 488-nm blue all-solid-state laser, a 640-nm red all-solid-state laser, and a photomultiplier tube. The fluorescence channels, including FITC, PE, PerCP, and APC, could all be monitored at the same time. NovoExpress software was used to complete data acquisition (ACEA Biosciences, China). The detection parameters included the all-channel area, width, height, and time, which could effectively distinguish adhered cells, cell debris, and single cells. A total of 20,000 events were examined in each sperm sample.

Semen aliquots were diluted in 1 × binding buffer to a concentration of 1.2 × 10^6^ cells/mL. Propidium iodide (PI, 5 μL, 50 μg/mL) was added to identify cell events, excluding cell debris, to assess sperm cells with plasma membrane damage using the protocol described by Bunel (18).

The sperm acrosome status was determined using FITC-peanut (Arachis hypogaea) agglutinin (GENMED SCIENTIFICS, INC., USA). Frozen-thawed sperm samples were diluted to 1.2 × 10^7^ cells/mL, mixed with 150 μL of PNA-FITC and 200 μL of PI (0.4 μg/mL), and incubated in the dark for 15 mins.

### 2.6. Metabolite extraction

To extract metabolites from 16 seminal plasma samples, 400 μL of cold extraction solvent (methanol/acetonitrile/H_2_O, 2:2:1, v/v/v) was added to 100-mg samples, which were then adequately vortexed. After vortexing, the samples were incubated on ice for 20 mins and then centrifuged at 14,000 × g for 20 mins at 4°C. The supernatant was collected and dried in a vacuum centrifuge at 4°C. For liquid chromatography–mass spectrometry (LC-MS) analysis, the samples were re-dissolved in 100 μL acetonitrile/water (1:1, v/v) solvent and transferred to LC vials. Quality control (QC) samples were created by pooling 10 μL of each sample and analyzing them with the other samples to evaluate the stability and repeatability of the instrument. The QC samples were assessed regularly, and one of every eight samples was evaluated.

### 2.7. LC-MS/MS analysis

The untargeted metabolomics of metabolic extracts were analyzed using a quadrupole time-of-flight mass spectrometer (Sciex TripleTOF 6600) using hydrophilic interaction chromatography *via* electrospray ionization. We used a gradient of solvent A (25 mM ammonium acetate in water and 25 mM ammonium hydroxide in water) and solvent B (acetonitrile) for chromatographic separation *via* an ACQUIY UPLC BEH Amide column (2.1 × 100 × 1.7 mm, Waters, Ireland). After holding at 85% B for 1 min, the gradient was linearly reduced to 65% over 11 mins, then reduced to 40% over 0.1 mins, held for 4 mins, and then increased to 85% over 0.1 mins, with a re-equilibration period of 5 mins. The following settings were used: flow rate of 0.4 mL/min, column temperature of 25°C, autosampler temperature of 5 °C, and injection volume of 2 μL. The mass spectrometer was operated in both negative and positive ionization modes. The electrospray ionization source conditions were set as follows: ion source gas 1: 60, ion source gas 2: 60, curtain gas: 30, source temperature: 600°C, ion spray voltage floating: ± 5,500 V. A rate of 0.20 s/spectra was set as the accumulation time for time-of-flight MS scans, with the instrument set to acquire over an m/z range of 60–1,000 Da. For automatic MS/MS acquisition, sample ion scans were acquired using information-dependent acquisition with high sensitivity mode and an m/z range of 25–1,000 Da. The accumulation time for product ions was set at 0.05 s/spectra. The parameters were set as follows: the collision energy was fixed at 35 V with ± 15 eV; declustering potential, 60 V (+) and −60 V (–); exclusion of isotopes within 4 Da; candidate ions to monitor per cycle, 10.

### 2.8. Data analysis

ProteoWizard MSConvert was used to convert the raw MS data (wiff.scan files) to MzXML files, which were then imported into the free version of XCMS software. The following parameters were used to select peaks: centWave m/z = 25 ppm and peak width = c (10, 19). The peak grouping parameters were bw = 5, mzwid = 0.025, and minfrac = 0.5. The processed data were uploaded before being imported into SIMCA-P (version 14.1, Umetrics, Umea, Sweden), where they were subjected to multivariate analysis, including Pareto-scaled principal component analysis (PCA) and orthogonal partial least squares-discriminant analysis (OPLS-DA). Variable importance in projection (VIP) values were calculated for each variable in the OPLS-DA model to determine their role in classification. A *t*-test was used to determine significance.

For Kyoto Encyclopedia of Genes and Genomes (KEGG) pathway annotation, the metabolites were searched against the online database. Fisher's exact test was used to conduct the KEGG pathway enrichment analysis. Pathways with *P*-values < 0.05 were considered significantly altered pathways.

### 2.9. Statistical analysis

The variables of all samples were analyzed *via* Student's *t*-test using SPSS statistical software (version 22.0; Chicago, IL, USA). Differences between the HF and LF samples were considered significant at a *P*-value < 0.05; data are presented as the mean ± SEM.

## 3. Results

### 3.1. Sperm quality evaluation

**Figure 1:**
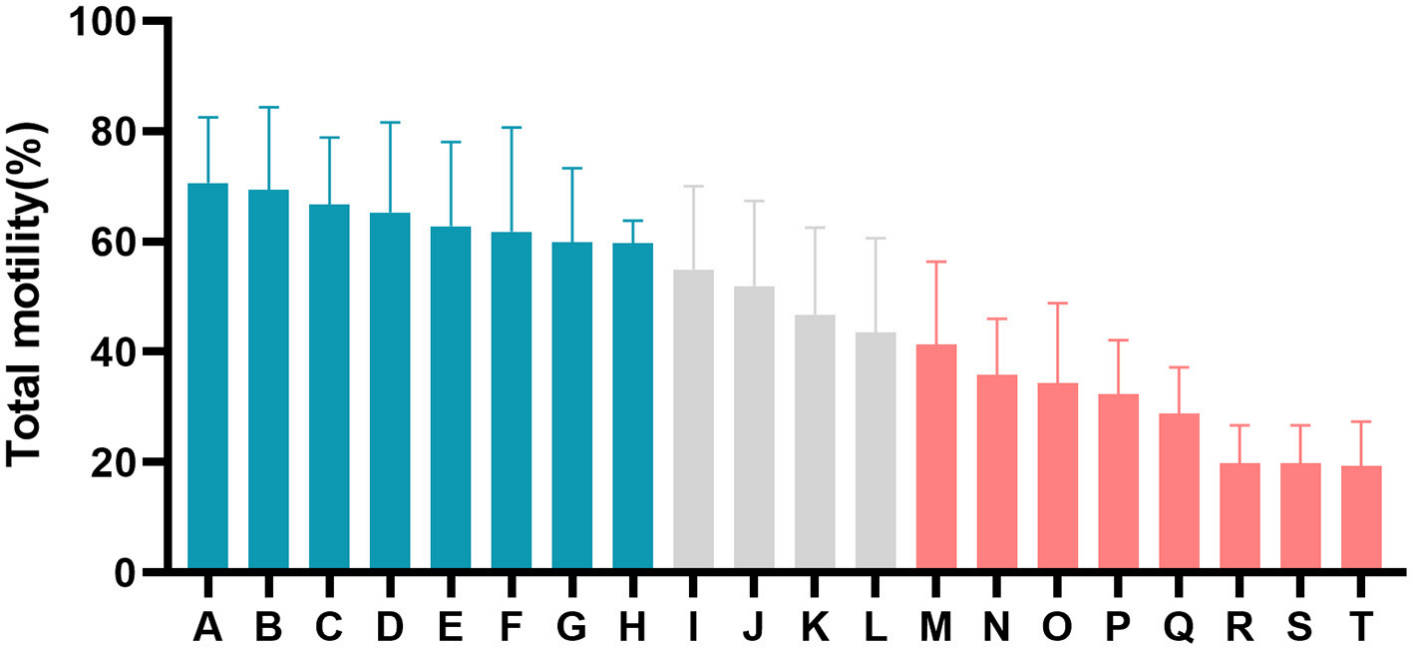
Difference in percentage total motility of post-thaw spermatozoa from all goats (A–T). The blue columns represent goats from the high-freezability (HF) group, the red columns represent goats from the low-freezability (LF) group, and the gray columns indicate goats with sperm classified as medium freezability.

The motion parameters of fresh semen showed no significant differences (*P* > 0.05, Table 1). An expected decline in total motility was observed in spermatozoa from each buck after the freezing-thawing process, but the degree of decline in total motility was different for the 20 goats (Figure 1; Table 1). The goats were divided into an HF group with >60% total motility (*n* = 8) and an LF group with <45% total motility (*n* = 8). CASA was conducted to evaluate various kinetic parameters, including TMOT, PMOT, VAP, VCL, VSL, ALM, and LIN, which were significantly different between the HF and LF groups (*P* < 0.001, Table 1). Spermatozoa from HF goats frozen with LSP displayed a significant decrease in sperm motility performance, and freezing of spermatozoa from LF goats with HSP had a significantly positive effect on the motion parameters (*P* < 0.001, Table 1).

**Table 1:** Differences in the motion kinematics of spermatozoa with high and low cryotolerance among fresh ejaculates, freeze-thaw, and EX-SP freeze-thaw samples.

**Characteristic**	**Fresh**		**Freeze-thawed**		**EX-SP freeze-thawed**	
	**HF**	**LF**	**F-HF**	**F-LF**	**EX-HF**	**EX-LF**
TM (%)	88.93 ± 0.9^a^	88.06 ± 1.29^a^	66.34 ± 5.3^b^	32.06 ± 2.53^c^	53.32 ± 6.16^d^	39.17 ± 4.48^e^
PM (%)	70.2 ± 4.24^a^	61.13 ± 5.25^b^	31.91 ± 3.12^c^	17.45 ± 1.77^d^	28.81 ± 3.86^c^	18.99 ± 6.55^d^
CLV (μm/s)	156.08 ± 24.7^a^	136.04 ± 12.08^b^	74.49 ± 6.20^c^	41.22 ± 9.75^d^	64.26 ± 11.01^c^	43.58 ± 11.73^d^
SLV (μm/s)	102.82 ± 21.6^a^	84.15 ± 15.93^b^	36.98 ± 2.75^c^	19.35 ± 3.11^d^	31.71 ± 5.11^c^	20.87 ± 5.81^d^
APV (μm/s)	115.30 ± 23.1^a^	98.49 ± 15.83^b^	45.21 ± 2.77^c^	23.76 ± 4.39^d^	39.16 ± 6.41^c^	25.53 ± 6.74^d^
ALH (μm)	5.44 ± 1.04^a^	6.17 ± 0.93^b^	3.83 ± 0.35^c^	2.23 ± 0.53^d^	3.69 ± 0.74^c^	2.42 ± 0.46^d^
LIN (%)	59.43 ± 9.37^a^	55.41 ± 9.11^a^	30.74 ± 2.67^b^	15.75 ± 3.43^c^	27.44 ± 4.05^b^	19.78 ± 3.59^c^

The different superscript letter indicate significant differences between the groups at *P* < 0.05.

**Figure 2:**
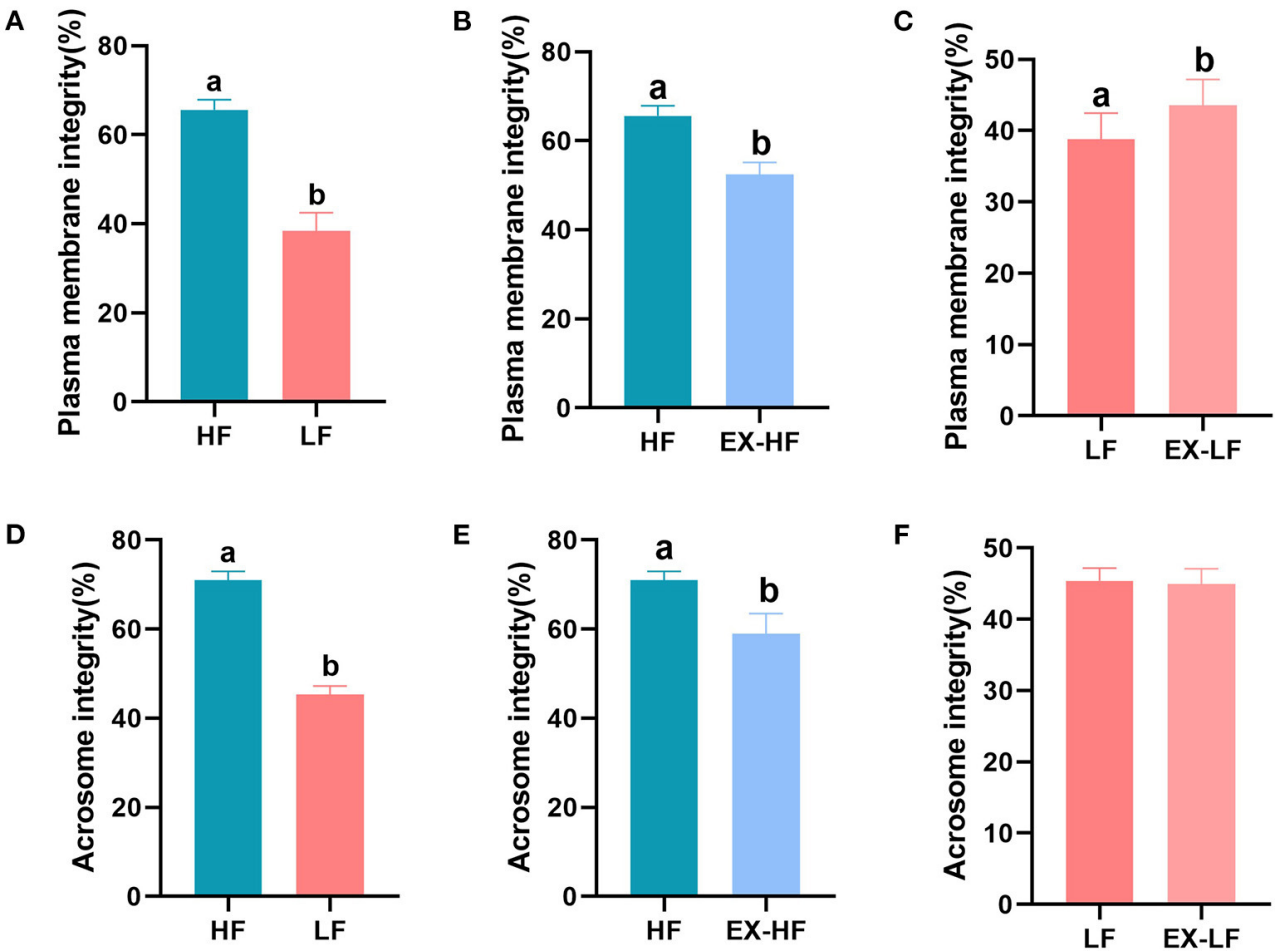
Percentage of sperm plasma membrane and acrosome integrity for goats with high and low freezing resilience. **(A–C)** Plasma membrane integrity for HF vs. LF, HF vs. EX-HF, and LF vs. EX-LF and **(D–F)** acrosome integrity for HF vs. LF, HF vs. EX-HF, and LF vs. EX-LF.

Significant differences in sperm plasma membrane and acrosome integrity between the HF and LF groups are shown in Figures 2A, D (*P* < 0.001). The acrosome integrity of spermatozoa from LF goats frozen with HSP was significantly increased (*P* < 0.001, Figure 2F), but plasma membrane integrity was not significantly affected (*P* > 0.05, Figure 2C). Spermatozoa from HF goats frozen with LSP exhibited a significant decline in plasma membrane and acrosome integrity (*P* < 0.001, Figures 2B, E).

### 3.2. Metabolic profile analysis

**Figure 3:**
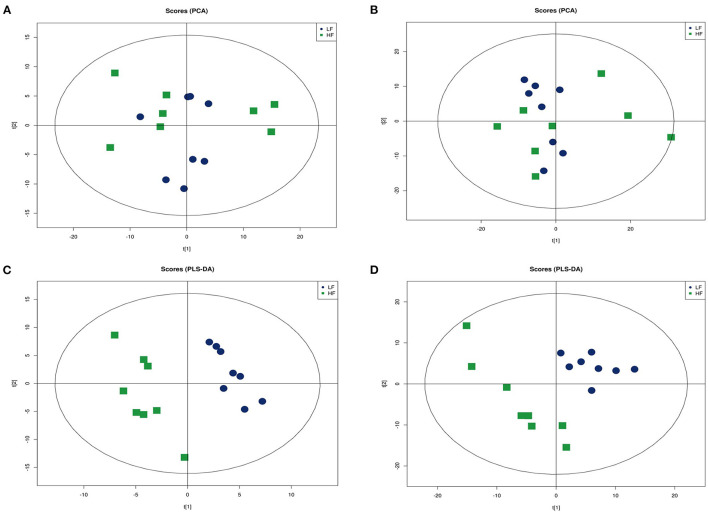
Multivariate statistical analysis of seminal plasma metabolic profiling of the HF and LF groups. **(A)** Principal component analysis (PCA) score plots in the positive ion mode. **(B)** PCA score plots in the negative ion mode. **(C)** Partial least squares-discriminant analysis (PLS-DA) score plot in the positive ion mode. **(D)** PLS-DA score plot in the negative ion mode.

An ultra-high performance liquid chromatography-quadrupole time-of-flight MS system (UHPLC-Q-TOF MS) was used to obtain metabolic profiles for seminal plasma from the HF and LF groups in the positive and negative ion modes. The peak intensity of each feature was obtained using XCMS software. In total, 9,408 and 9,342 molecular features were extracted from each sample in the positive and negative ion modes, respectively. To identify ion peaks that could be used to distinguish between the metabolite profiles of seminal plasma from the HF and LF groups, we used subsequent analytical models to determine the best fit that may reflect changes in categorical identification differences. We plotted the unsupervised PCA scores of all seminal plasma profiles from the HF and LF groups. However, the results showed no intrinsic clustering related to semen freezability in the first two PCs. The PCA score chart showed that QC samples clustered together, indicating that the instrument was stable during the entire sample collection process. Although partial overlap was observed between the HF and LF groups, an overall trend toward separation was noted, both in the positive and negative ion modes (Figures 3A, B). Next, PLS-DA was performed on identified metabolites of seminal plasma from the HF and LF groups under both positive and negative ion modes to determine the significant variables among the different samples (Figures 3C, D). The results indicated a separation of clusters in PLS-DA plots for samples from the two groups. The separation of clusters indicated that significant differences existed among the analyzed samples for both the positive and negative ion modes.

### 3.3. Screening and identification of differentially expressed metabolites

Based on the successful discrimination of seminal plasma from the HF and LF groups, a search for the specific metabolites that contributed to the metabolomic differences between the two groups was conducted. VIP values were used to evaluate the influence of the expression pattern of each metabolite on the classification and discrimination of samples. Potential differentially-expressed metabolites with a VI *P*-value > 1 and 0.05 < *P*-value < 0.1 were selected as differential metabolites, and metabolites with a VI *P*-value > 1 and *P*-value < 0.05 were selected as significant differential metabolites. Then, 38 discriminating differential metabolites were identified in seminal plasma, including 21 in the positive ion mode and 17 in the negative ion mode (Tables 2, 3).

**Table 2:** Differential metabolites between the LF and HF groups in the negative ion mode.

**HMDB**	**Description**	**VIP**	**FC**	***P*-value**
HMDB0000094	Citrate	12.43	1.28	0.0842
HMDB0000695	Ketoisocaproic acid	10.54	2.09	0.0083
HMDB0000641	L-Glutamine	7.82	0.64	0.0031
HMDB0004049	20-Hydroxy-PGF2a	4.96	0.45	0.0542
HMDB0001320	15-Keto-PGE1	4.64	0.65	0.0810
HMDB0000210	Pantothenate	3.86	1.66	0.0936
HMDB0000207	Oleic acid	3.53	0.55	0.0464
HMDB0000079	Dihydrothymine	3.34	0.63	0.0025
HMDB0000673	Linoleic acid	2.44	1.62	0.0818
HMDB0000462	Allantoin	1.90	1.35	0.0195
HMDB0002340	2-Methylbenzoic acid	1.67	1.90	0.0236
HMDB0001858	p-Cresol	1.53	2.45	0.0477
HMDB0000205	Phenylpyruvate	1.44	1.71	0.0059
HMDB0001870	Benzoic acid	1.41	1.49	0.0067
HMDB0000191	L-Aspartate	1.25	1.45	0.0225
HMDB0001311	DL-lactate	1.20	0.72	0.0839
HMDB0003345	Alpha-D-Glucose	1.13	1.69	0.0811

**Table 3:** Differential metabolites between the LF and HF groups in the positive ion mode.

**HMDB**	**Description**	**VIP**	**FC**	***P* value**
HMDB0029011	Pro-Arg	9.61	1.30	0.0742
HMDB0000201	Acetylcarnitine	8.26	1.46	0.0147
HMDB0000157	Hypoxanthine	7.73	1.17	0.0891
HMDB0000267	L-Pyroglutamic acid	6.82	0.64	0.0006
HMDB0000687	L-Leucine	4.25	1.36	0.0859
HMDB0000306	Tyramine	4.09	1.43	0.0777
HMDB0000159	L-Phenylalanine	3.48	1.44	0.0713
HMDB0000177	L-Histidine	3.00	1.37	0.0917
HMDB0000641	L-Glutamine	2.97	0.70	0.0002
HMDB0000097	Choline	2.88	0.75	0.0422
HMDB0000062	L-Carnitine	2.77	0.78	0.0260
HMDB0029030	Pro-Val	1.98	1.76	0.0921
HMDB0000848	Stearoylcarnitine	1.86	2.71	0.0022
HMDB0000210	Pantothenate	1.79	1.64	0.0790
HMDB0001403	PGD2	1.44	0.56	0.0051
HMDB0003357	N2-Acetyl-L-ornithine	1.25	0.67	0.0402
HMDB0036458	1-Aminocyclopropane carboxylic acid	1.22	0.66	0.0203
HMDB0000517	L-Arginine	1.12	0.75	0.0741
HMDB0003229	cis-9-Palmitoleic acid	1.09	0.70	0.0054
HMDB0000230	N-Acetylneuraminic acid	1.06	1.53	0.0244
HMDB0000191	L-Aspartate	1.05	1.52	0.0898

### 3.4. Analysis of metabolic pathways

**Figure 4:**
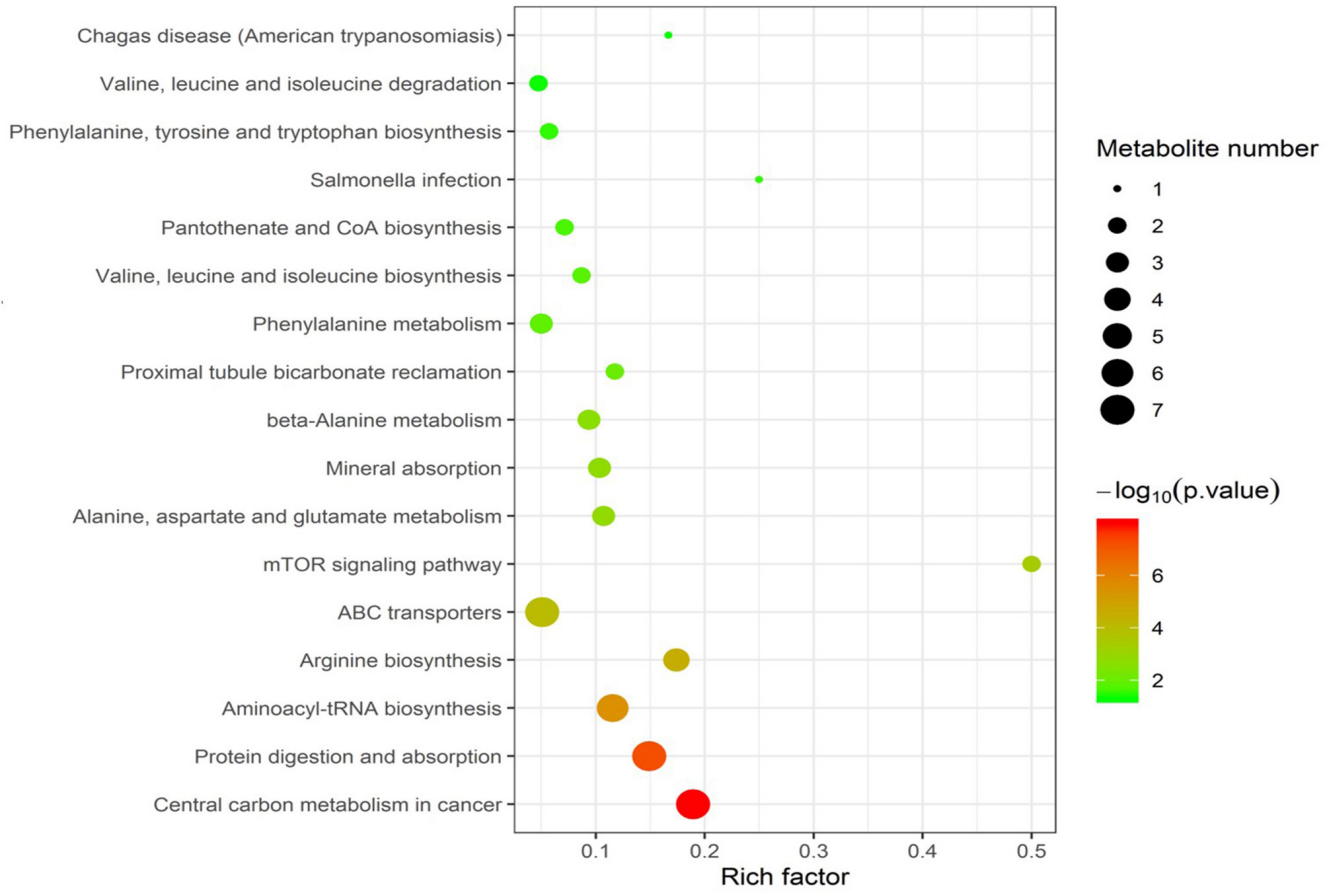
Kyoto Encyclopedia of Genes and Genomes enrichment of differential metabolites.

**Figure 5:**
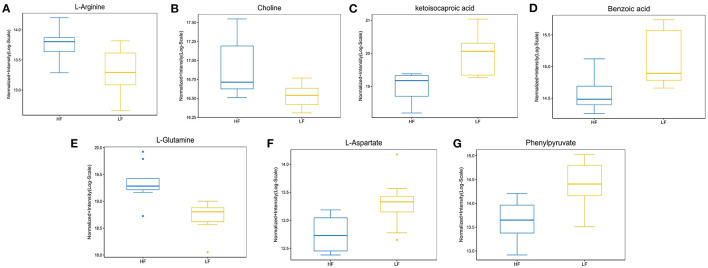
The abundance ratio of important metabolites in HF and LF goat seminal plasma. **(A)** L-arginine, **(B)** choline, **(C)** ketoisocaproic acid, **(D)** benzoic acid, **(E)** L-glutamine, **(F)** L-aspartate, and **(G)** phenylpyruvate showed significantly different abundance between HF and LF goat seminal plasma.

**Figure 6:**
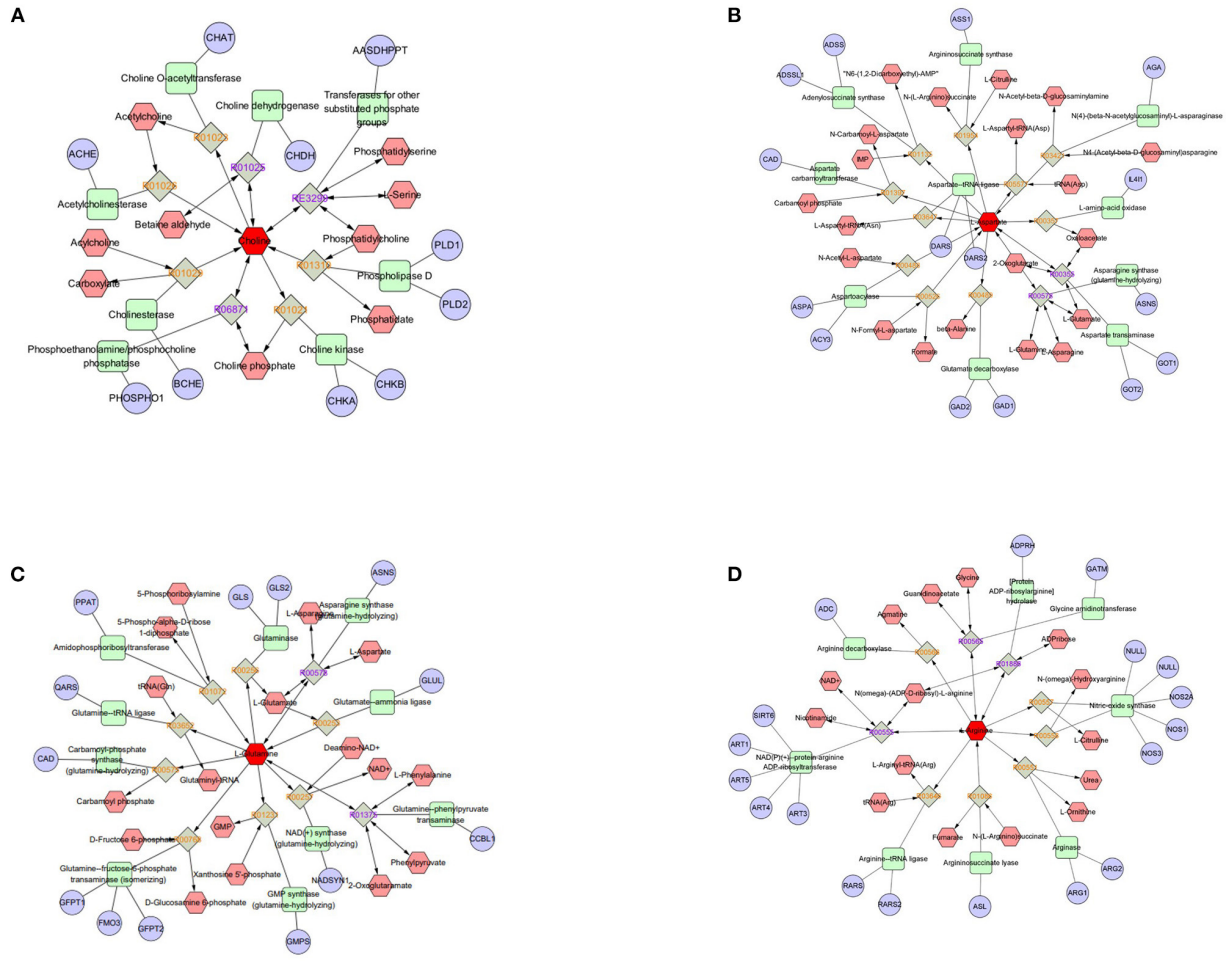
Compound-reaction-enzyme-gene networks of important differential metabolite biomarkers de-termined using Metscape. **(A)** Choline, **(B)** L-aspartate, **(C)** L-glutamine, and **(D)** L-arginine. The metabolites are shown in red hexagons. Gray squares: reaction nodes with reaction ID; pale red hexagons: compound nodes; green squares: enzyme nodes; blue circles: gene nodes.

**Figure 7:**
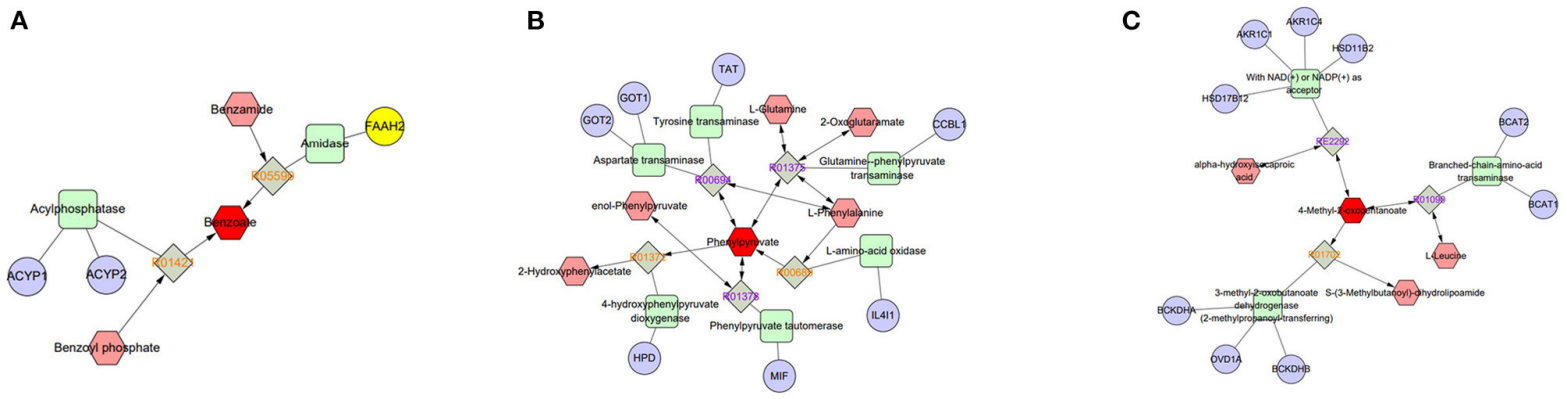
Compound-reaction-enzyme-gene networks of important differential metabolite biomarkers de-termined using Metscape. **(A)** Benzoic acid, **(B)** phenylpyruvate, and **(C)** 4-methyl-2-oxopentanoate (ketoisocaproic acid). The metabolites are shown in red hexagons. Gray squares: reaction nodes with reaction ID; pale red hexagons: compound nodes; green squares: enzyme nodes; blue circles: gene nodes.

Enrichment pathway analysis was performed using Fisher's exact test and the KEGG metabolic pathway database to identify differentially-expressed metabolites. Statistically significant pathways that were overrepresented included central carbon metabolism in cancer; protein digestion and absorption; aminoacyl-tRNA; arginine biosynthesis; ABC transporters; mTOR signaling pathway; alanine, aspartate, and glutamate metabolism; mineral absorption; β-alanine metabolism; proximal tubule bicarbonate reclamation; phenylalanine metabolism; valine, leucine, and isoleucine biosynthesis; pantothenate and CoA biosynthesis; salmonella infection; phenylalanine, tyrosine, and tryptophan biosynthesis; valine, leucine, and isoleucine degradation; and Chagas disease (Figure 4). The enrichment pathway indicated seven differential metabolites, including L-glutamine, L-aspartate, L-arginine, phenylpyruvate, benzoic acid, ketoisocaproic acid, and choline; their abundance was significantly different between seminal plasma from the HF and LF groups (Figures 5–7).

## 4. Discussion

Semen freezability was affected by several factors, including the components of the freezing extender, cryopreservation procedure, lipid composition of sperm, and regulation effect of seminal plasma (20–22). In this study, semen from different individual goats presented diverse degrees of cryodamage, including a remarkable reduction in sperm motility and structural integrity after thawing. Our results demonstrated that seminal plasma makes an important contribution to sperm freezability. Furthermore, HSP significantly improved the post-thaw quality of LF spermatozoa, while LSP decreased the motility parameters and structural integrity of thawed spermatozoa with HF. Several studies have demonstrated the beneficial effects of seminal plasma on sperm cryopreservation. Sperm frozen with HSP exhibited superior motility and cell structural integrity after thawing (9, 23–25). Thus, the variation in freezability of goat semen is associated with differences in seminal plasma composition.

Seminal plasma provides sperm with buffering and energy materials, and small metabolic molecules in seminal plasma are involved in regulating important reproductive events of sperm, such as sperm motility, capacitation, the acrosome reaction, fertilization, and embryo development (26). Metabolomic analysis enables the systematic study of small-molecule metabolites that respond to downstream events in gene expression and protein regulation that are closely related to the functional phenotype of the cell. Additionally, metabolomic analysis of key biomarkers can be used to determine the relationship between metabolite expression patterns and the cellular phenotype (27). In this study, we examined seminal plasma of the HF and LF groups using metabolomic analysis, and PLS-DA revealed a significant separation of the metabolic profiles of the two groups in positive and negative ion patterns. Similar results were found for donkey and pig seminal plasma, i.e., significant differences in the expression patterns of seminal plasma metabolites with different freezing resistance (28, 29). Bioinformatics analysis revealed that the metabolites enriched according to KEGG pathway analysis were significantly different between the HF and LF groups, including L-glutamine, L-aspartate, L-arginine, choline, phenylpyruvate, benzoic acid, and ketoisocaproic acid, which are involved in important physiological functions and amino acid biosynthesis in goat spermatozoa.

The diverse amino acids found in seminal plasma have a variety of biological functions, including reducing free radicals and protecting cells from degeneration (30). The amino acid profiles of bovine seminal plasma with different freezing resistances were clearly distinct and could be used to identify the freezing resistance phenotype (31). Specific amino acids in chicken seminal plasma are associated with sperm viability and DNA integrity after thawing (32). Glutamine is a potent antioxidant in semen, and several studies have found that glutamine protects sperm from reactive oxygen species by enhancing glutathione synthesis (33). Glutamine is a component of sperm cryodilution for several species, such as rabbits (33), boars (34), bucks (35), rams (36), bulls (37), and mouse (38). L-arginine can improve sperm motility and mitochondrial activity after thawing and reduce the structural damage to sperm (39–42). In addition, arginine can increase the proportion of capacitated sperm after thawing. One effective method to produce blastocysts is to add an appropriate amount of arginine during *in vitro* fertilization (43, 44). The addition of aspartate to the medium can protect sperm from oxidative stress damage and plays a positive role in maintaining sperm vitality and reducing lipid peroxidation and DNA fragmentation. Additionally, the developmental ability of bovine sperm embryos treated with aspartic acid was significantly improved (45, 46). Oral aspartate was shown to improve the fertility of young male C5BL/6N mice *in vivo*, increase freeze-thaw sperm quality in sexually immature mice, and play a direct role in the sperm capacitation process and acrosome reaction (47–49).

As a consequence of the extensive protein hydrolysis activity occurring in semen, the concentration of many amino acids increases after ejaculation, and oxidizable substrates capable of acting as energy supplies cause reactions (50, 51). Phenylalanine was identified in bovine seminal plasma and can be considered a biomarker of freezability (31). Moreover, Zhang described the positive effect of leucine on sperm motility in zebrafish (52). Additionally, the concentration of leucine was significantly reduced in the sperm of low-fertility bulls (14). Although a difference in the abundance of phenylalanine and leucine in seminal plasma was found between the two groups in this study, it was not statistically significant. However, the levels of benzoic acid, phenylpyruvate, and ketoisocaproic acid, as intermediate products of phenylalanine and leucine anabolic pathway processes, were significantly different between the HF and LF goat seminal plasma groups in this study, which may indicate a correlation between phenylalanine and leucine anabolism of goat spermatozoa and semen freezability. Menezes compared the metabolic characteristics between bovine spermatozoa with high and low fertility and found remarkable differences in benzoic acid abundance ratios (53). As an intermediate in the anabolic pathway of phenylalanine, the pheA gene encodes a functional enzyme that rearranges chorismate to prephenate and then converts it into phenylpyruvate (54). Benzoic acid is biosynthesized from water-soluble phenylalanine *via two* non-oxidative pathways and a CoA-dependent β-oxidative pathway (55). Spermatogenic cells are equipped to produce ketoisocaproic acid and lactic acid from leucine and glucose using supporting cells (56).

Choline is an important precursor for the synthesis of phosphatidylcholine (PC), which is produced in animal cells *via* the CDP-choline pathway. Phosphatidylethanolamine can be converted into PC in cells, which is then catabolized to choline (57). PPC is an essential phospholipid in mammalian cells and tissues, and several studies have illustrated the importance of PC for sperm freezability, including the ability of PC to affect sperm motility and fertilization ability (20, 58, 59). PC is a major component of cryoprotectants during the semen cryopreservation process of several species, and substances such as egg yolk and soy lecithin prevent decreases in sperm viability and structural integrity after thawing (19, 60).

## 5. Conclusions

In summary, wide individual variability was observed in goat semen freezability, and the composition of seminal plasma may affect sperm freezability. In this study, 41 differential metabolites were identified between goat seminal plasma with high and low semen freezability by metabolomic analysis. Therefore, sperm freezability may be directly affected by amino acids in goat seminal plasma, such as L-glutamine, L-aspartate, and L-arginine. In addition, intermediate metabolites in the anabolic processes of phenylalanine, leucine, and phosphatidylcholine, including phenylpyruvate, benzoic acid, ketoisocaproic acid, and choline, may indirectly regulate the cryotolerance of sperm, and these metabolites may serve as potential biomarkers of goat sperm freezability.

## Data availability statement

The datasets presented in this study can be found in online repositories. The names of the repository/repositories and accession number(s) can be found in the article/supplementary material.

## Ethics statement

The animal study was reviewed and approved by the Academic Ethics Committee of Inner Mongolia Agricultural University [Approval No: (2020) 056]. Written informed consent was obtained from the owners for the participation of their animals in this study.

## Author contributions

BX conceptualized the study and wrote the manuscript. BX, JL, and JiaxZ conceptualized, designed, and carried out the investigations. BX, XB, JianZ, and BL performed the experiments. BX, YZ, RS, RW, ZW, and QL analyzed the data. JiaxZ and JL critically revised the manuscript. All authors read and approved the final manuscript.
